# Successful management of belimumab after obinutuzumab in a patient with systemic lupus erythematosus: a case report with an 18-month follow-up

**DOI:** 10.3389/fimmu.2024.1459241

**Published:** 2024-10-03

**Authors:** Xiuxiu Pu, Qiao Ye, Lin Zhu, Tingting Yan

**Affiliations:** ^1^ Jiaxing University Master’s Degree Cultivation Base, Zhejiang Chinese Medical University, Jiaxing, China; ^2^ Department of Rheumatology, The Second Affiliated Hospital of Jiaxing University, Jiaxing, China

**Keywords:** systemic lupus erythematosus, lupus nephritis, gastrointestinal involvement, obinutuzumab, belimumab

## Abstract

**Introduction:**

Systemic lupus erythematosus (SLE) is a complex autoimmune disease, and despite the availability of multiple treatments, striking a balance between long-term efficacy and side effects remains a major clinical challenge. B-cell–directed therapy has attracted much attention because of its unique mechanism of action. Belimumab and obinutuzumab, as representative drugs for B-cell–directed therapy, have shown their respective advantages for SLE treatment. However, data on combination therapy with obinutuzumab and belimumab are currently unavailable.

**Case presentation:**

We present the severe case report of a patient who was diagnosed with lupus nephritis (LN) with gastrointestinal involvement and developed acute renal failure. The patient responded to the first dose of obinutuzumab but failed to achieve a complete response to LN. The repeated use of obinutuzumab was limited by persistently low IgG levels and frequent infections. This is a real-world challenge that must be addressed. Therefore, the patient was subsequently treated with a novel sequential regimen of obinutuzumab followed by belimumab. After 18 months of follow-up, the patient achieved a complete clinical response with a favourable safety profile, along with the conversion of all autoantibodies from positive to negative and sustained negativity. To date, the patient has achieved a dual clinical and serological response.

**Conclusion:**

There is a reason to believe that this novel combination regimen could be developed as a therapeutic strategy, with the expectation of balancing efficacy and safety.

## Introduction

Cell-directed therapies are at the forefront of systemic lupus erythematosus (SLE) treatment. The range of targets and drug species being studied are rich, diverse, and numerous (1, 2). Belimumab, a biologic B-cell activating factor inhibitor (BAFF), was the first biologic agent licenced for SLE therapy thus far (3). Obinutuzumab is a humanised type II anti-CD20 monoclonal antibody directed at a different epitope on CD20 than that bound by belimumab, resulting in a profound B-cell apoptotic response and CD20 depletion. The Food and Drug Administration granted this breakthrough therapy status to obinutuzumab after achieving responses in the NOBILITY clinical trial for lupus nephritis (LN) (4). Although belimumab and obinutuzumab each have advantages in the treatment of SLE, there are currently no reports on the combined use of these two biologic agents.

Notably, rituximab is the first anti-CD20 monoclonal antibody used worldwide. However, because the EXPLORER and LUNAR trials failed to achieve the primary clinical endpoint in SLE patients, rituximab remains an off-label drug for SLE (5, 6). A randomized controlled trial (RCT) confirmed that the administration of belimumab after rituximab significantly reduces serum immunoglobulin G (IgG) anti–ds-DNA antibody levels and decreases the risk for severe flares in patients with SLE refractory to conventional therapy (7). As an improved version of rituximab, obinutuzumab has a stronger binding ability to the CD20 antigen and B-cell depletion capacity (8). Therefore, it is necessary to explore the potential of the combined use of belimumab and obinutuzumab for the treatment of SLE. Here, we report the case of a patient with severe SLE manifesting with LN and gastrointestinal involvement who was successfully treated with belimumab after obinutuzumab and discuss the feasibility of such a novel combination regimen.

## Case presentation

In September 2022, a 62-year-old woman with continuous abdominal pain and vomiting for 7 days came to our hospital. The patient presented with upper abdominal pain, frequent vomiting, and fever, with a temperature ranging from 37.5°C to 38°C. She felt that her urine volume had decreased; however, there were no noticeable changes in her urine or colour, and she denied having joint pain, rashes, or oedema. The patient had hypertension and denied a history of digestive system diseases. In addition, she did not smoke or drink alcohol, did not use addictive drugs, and had no family history of autoimmune diseases or genetic disorders. Her blood pressure was 150/78 mmHg, and all other vital signs were normal. There was tenderness in her upper abdomen. Computed tomography (CT) revealed widespread oedema and thickening of the small intestinal wall, multiple enlarged lymph nodes in the abdomen, and no signs of cirrhosis. Laboratory test results from the beginning of hospitalization are shown in **Table 1**.

**Table 1:** Laboratory results of the patient before receiving belimumab treatment.

Blood cell count	Result		Urinary specific gravity	1.014	IgM	1.45 g/L
White blood cells	8.49 × 10^9/L		Urinary occult blood	1+ (↑)	IgG4	0.692 g/L
Neutrophils	7 × 10^9/L (↑)		Urinary-Whitebloodcell	Negative	T cells (CD3+)	50.00% (↓)
Monocytes	0.51 × 10^9/L		Urinary-Redbloodcell	Negative	T suppressor cells (CD8+/CD3 +)	32.00%
Lymphocytes	0.97 × 10^9/L (↓)		Urinary glucose	Normal	T helper cells (CD4+/CD3 +)	17.00% (↓)
Red blood cells	4.55 × 10^9/L		Urinary protein	3+ (↑)	CD4/CD8	0.53 (↓)
Haemoglobin	76 g/L (↓)		Urinary cast	Negative	NK cells (CD3-/CD16 + 56+)	8.00%
Platelets	149 × 10^9/L		24-h urine total protein	0.3 g (↑)	B cells (CD19+)	42.00% (↑)
Biochemical	Result		Immunological tests	Result	Absolute CD19+ B-cell count	430 cells/µl
Total bilirubin	5.4 μmol/ml		Antinuclear antibody titres	1:1000 (↑)	C-reactive protein	<0.80 mg/L
Direct bilirubin	3.4 μmol/ml		Anti-Smith antibody	Positive (↑)	Erythrocyte sedimentation rate	6 mm/H
Indirect bilirubin	2.0 μmol/ml		Anti-nucleosome antibody	Positive (↑)	Others	Result
Total bile acid	0.20 μmol/L		Anti-histone antibody	Positive (↑)	Procalcitonin	0.108 ng/ml (↑)
Total protein	70.4 g/L (↓)		Anti–ds-DNA antibody	Positive (↑)	D-dimer	>4400 μg/L (↑)
Albumin	25.6 g/L (↓)		Anti–ds-DNA antibody titres	223 IU/ml (↑)	B-type natriuretic peptide	121.09 pg/ml (↑)
Globulin	44.8 g/L (↑)		Anti-ribosomal P-protein antibody	Positive (↑)	Deterioration of renal function	Result (peak)
Alanine aminotransferase	15 U/L		Anti-mitochondrial antibody M2 subtype	Positive (↑)	Serum creatinine	263 μmol/L (↑)
Aspartate aminotransferase	62 U/L (↑)		C-type anti-neutrophil cytoplasmic antibody	Negative	Serum β2-microglobulin	6.17 mg/L (↑)
γ-Glutamyltranspeptidase	38 U/L		P-type anti-neutrophil cytoplasmic antibody	Positive (↑)	Urinary pH	5.5
Alkaline phosphatase	65 U/L		PR3 anti-neutrophil cytoplasmic antibody	Negative	Urinary specific gravity	1.015
Lactate dehydrogenase	299 U/L (↑)		MPO anti-neutrophil cytoplasmic antibody	Negative	Urinary occult blood	2+ (↑)
Total cholesterol	6.02 mmol/L (↑)		ADAMTS13 activity	26.31%	Urinary white blood cells	65 cells/hpf (↑)
Triglycerides	1.52 mmol/L		ADAMTS13 activity inhibitory antibody	223 IU/ml (↑)	Urinary red blood cells	249 cells/hpf (↑)
Serum ferritin	410.5 ng/ml (↑)		Anti-glomerular basement membrane antibody	Negative	Urinary glucose	Normal
Uric acid	491.5 μmol/L (↑)		Anti-cardiolipin IgG antibody	Positive	Urinary protein	3+ (↑)
K	3.57 mmol/L		anti-β2GP1 IgG antibody	Positive	Urinary cast	Negative
Na	142.7 mmol/L		Complement 3	0.24 g/L (↓)	24-h urine total protein	12.3 g (↑)
Cl	109.1 mmol/L		Complement 4	0.04 g/L (↓)	Urinary a1-microglobulin	112 mg/L (↑)
Ca	1.86 mmol/L (↓)		Rheumatoid factor	45.3 IU/ml (↑)	Urinary microalbumin	>1000 mg/L (↑)
P	1.56 mmol/L(↑)		Coombs test	Negative	Urinary transferrin	> 9 0 mg/L (↑)
Urine analysis			IgG	21.30 g/L (↑)	Urinary IgG	>200 mg/L (↑)
Urinary PH	5.0		IgA	5.4 g/L (↑)		

The patient was suspected of having infectious gastroenteritis, and her abdominal pain did not resolve after antibiotic treatment. The possibility of autoimmune disease involving the gastrointestinal tract was considered. Further laboratory results revealed high ANA titres (1:1000) and widespread positive immunoblotting of autoantibodies, including anti-Smith, anti-nucleosome, anti-histone, anti–ds-DNA, anti-ribosomal P-protein, and anti-mitochondrial M2 subtype antibodies. Anti–ds-DNA antibody titres were measured by chemiluminescence up to 224 (IU/ml). In addition, anti-cardiolipin IgG antibodies and anti-β2GP1 IgG antibodies were both positive. Then, she underwent a thorough inquiry regarding her medical history. She told the doctor that she had been diagnosed with SLE 5 years prior, after being examined for dry mouth and severe dental problems, and that she had briefly (for 1 month) taken hydroxychloroquine. However, she refused to take glucocorticoids. She believed that she was healthy and did not see SLE as a threat to her health. After evaluation, we concluded that she met the diagnostic classification criteria for SLE. She began treatment with an IV of 80 mg/day methylprednisolone, 0.1 g of hydroxychloroquine orally twice daily, and nadroparin calcium 4100AXaIU via subcutaneous injection once daily, and her abdominal pain improved slightly.

**Figure 1:**
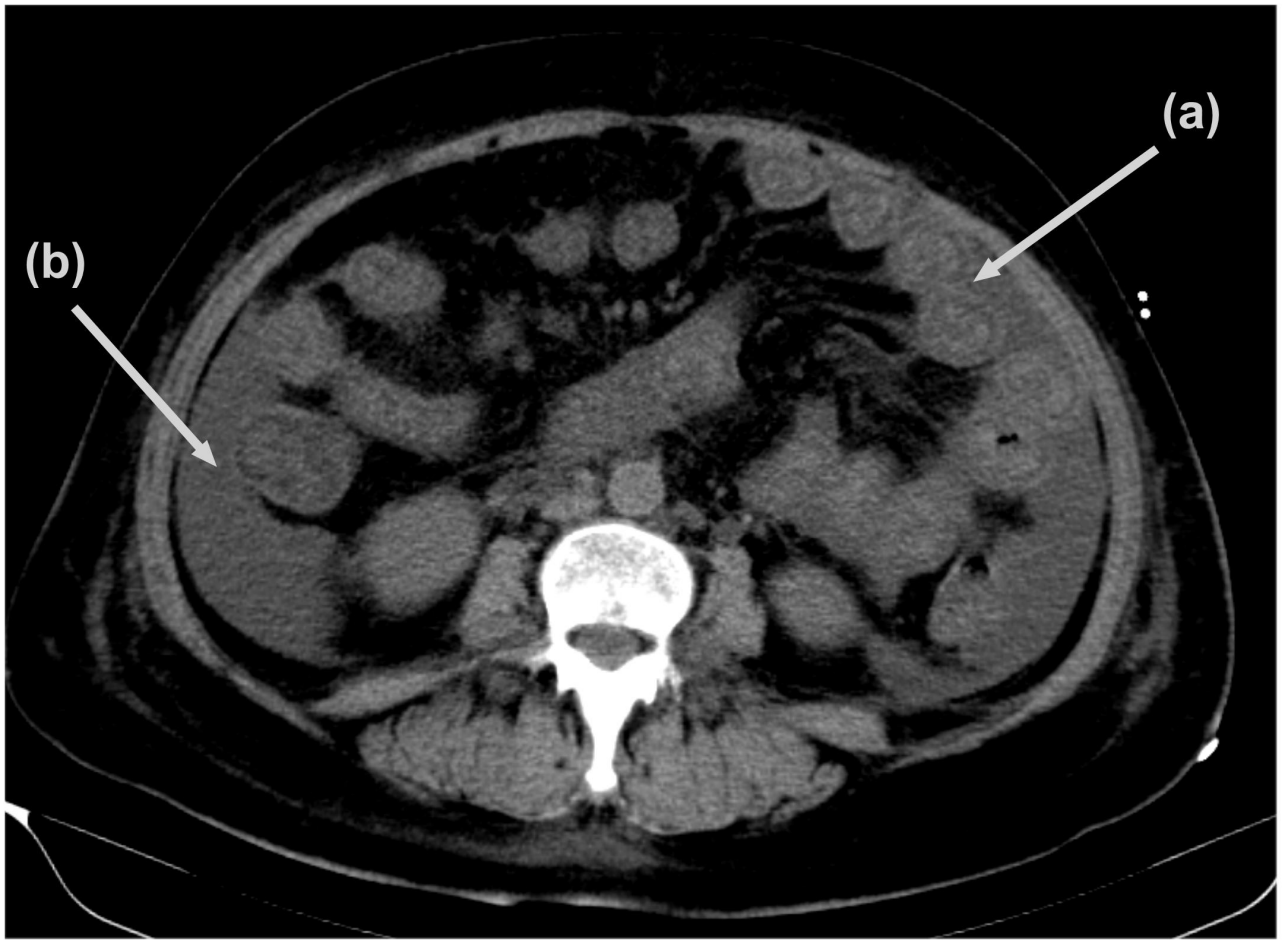
Abdominal computerized tomography findings. (a) Diffuse bowel wall thickening, also referred to as the doughnut sign or target sign; (b) massive peritoneal effusion.

On hospital day 5, the patient suddenly felt tightness and wheezing in her chest, which was severe, and she was unable to lie flat. Additionally, she exhibited a marked increase in her abdominal circumference, positive shifting dullness, and significant swelling in the buttocks, sacrum, and lower limbs. Upon re-evaluation of the CT scan, bilateral pleural effusion and massive pelvic effusion were identified. Additionally, extensive abdominal intestinal wall oedema and thickening with target signs (**Figure 1**), as well as extensive subcutaneous exudation of the abdominal wall, were noted. Multiple enlarged lymph nodes were observed in various regions, including the mediastinum, bilateral axilla, right cardiophrenic angle, splenic portal area, retroperitoneum, ileocecal region, bilateral iliac vessels, and inguinal region. The laboratory results revealed that her albumin was only 25.6 g and her 24-h urine total protein (24UTP) was 0.3 g. Based on specific immunological markers and gastrointestinal imaging, we concluded that the diagnosis of SLE was reasonable. Typical target signs on CT images revealed mesenteric vasculitis in SLE patients. In addition, protein-losing gastroenteropathy was also considered due to significant oedema and hypoalbuminaemia. These findings are suggestive of severe activity in SLE patients. Subsequently, her glucocorticoid therapy was adjusted to 200 mg/day methylprednisolone, and 20 g/day intravenous immunoglobulin (IVIG) was also administered for 2 days as supplementary therapy for severe SLE and after high doses of glucocorticoids were administered. On methylprednisolone pulse treatment day 3, the patient’s 24-h urine output was only 750 ml. Her creatinine level was 263.24 μmol/L. Her 24UTP was 8.8 g, and a reassessment the next day revealed an increase to 12.3 g. Routine urine examination revealed 260 red blood cells and 65 white blood cells. On the basis of these clinical findings, LN was diagnosed.

As a renal manifestation of SLE, LN is usually a chronic, progressive process, and acute renal failure (ARF) is not a typical manifestation. The patient’s phospholipid antibody positivity and significantly elevated D-dimer levels were considered for the possibility of secondary antiphospholipid syndrome in SLE, which could increase the risk of renal artery thrombosis. In addition, we also checked the patient’s disintegrin and metalloproteinase with a thrombospondin type 1 motif, member 13 (ADAMTS13) activity, which was 26.31%, and found that she was negative for ADAMTS13 inhibitory antibody. These could help in differential diagnosis. However, owing to the large amount of ascites in the abdomen of the patient at that time, kidney puncture could not be performed, and the definite pathological diagnosis of the kidney could not be clarified. To ensure an effective blood volume, we guided fluid rehydration by monitoring CVP, supplementing with albumin, facilitating the return of interstitial fluid to blood vessels, and administering diuretics. We observed a gradual increase in urine volume, a gradual recovery of creatinine, and improvement in symptoms of shortness of breath. These findings suggested that the cause of ARF was likely to be prerenal, with hypoproteinaemia and massive effusion resulting in insufficient effective blood volume or significantly enlarged abdominal lymph nodes pressing on the renal artery. The patient’s SLE disease activity index score of 19 indicated severe disease (fever, erythrocyturia, proteinuria, pyuria, pleurisy, low complement, and elevated anti–ds-DNA). Notably, gastrointestinal injury is not included in the SLEDAI. Because her SLE was highly active, the digestive tract and kidneys were severely damaged, and the disease progressed rapidly. She had 42% CD19+ B cells and a highly active immune state. CD20 depletion therapy is considered promising for quickly achieving control of the immune storm and preventing further organ damage. Obinutuzumab was chosen because it is a fully humanized antibody that is less immunogenic and causes fewer allergic reactions and greater CD20 depletion capacity.

On 21 September 2022, the patient received the first dose of 1000 mg per day of obinutuzumab. The process went smoothly, and the patient felt no discomfort. One week later, her absolute B-cell count decreased to one. She was scheduled to receive a second infusion of obinutuzumab on day 14; however, she developed an upper respiratory infection, manifested as a severe cough and catarrhal rhinitis. Her chest CT re-examination revealed no signs of pneumonia. She was treated with 40 mg/day methylprednisolone; however, her temperature remained above 38°C, and her white blood cell count decreased to 2.93 × 10^9/L. Consequently, she underwent bone marrow aspiration and biopsy. She subsequently received an infusion of 10 g of IVIG. After 3 days, her temperature gradually normalized, and her cough improved. No abnormal cell proliferation or infiltration was found in the bone marrow. She was discharged on 15 October 2022 with glucocorticoid therapy adjusted to 40 mg/day prednisone. Abdominal pain and the ability to eat returned, but her ankles and calves remained swollen. The 24UTP was 7.2 g, so to reduce proteinuria more effectively, the patient started taking 1.5 g/day mycophenolate morphenate (MMF). Upon re-examination on 3 November 2022, her antinuclear antibodies were positive (1:1000), and immunoblotting for all the other extractable nuclear antigen antibodies, including anti–ds-DNA antibodies, was negative. This finding was encouraging, although we determined an anti–ds-DNA antibody titre of 113 IU/ml.

**Figure 2:**
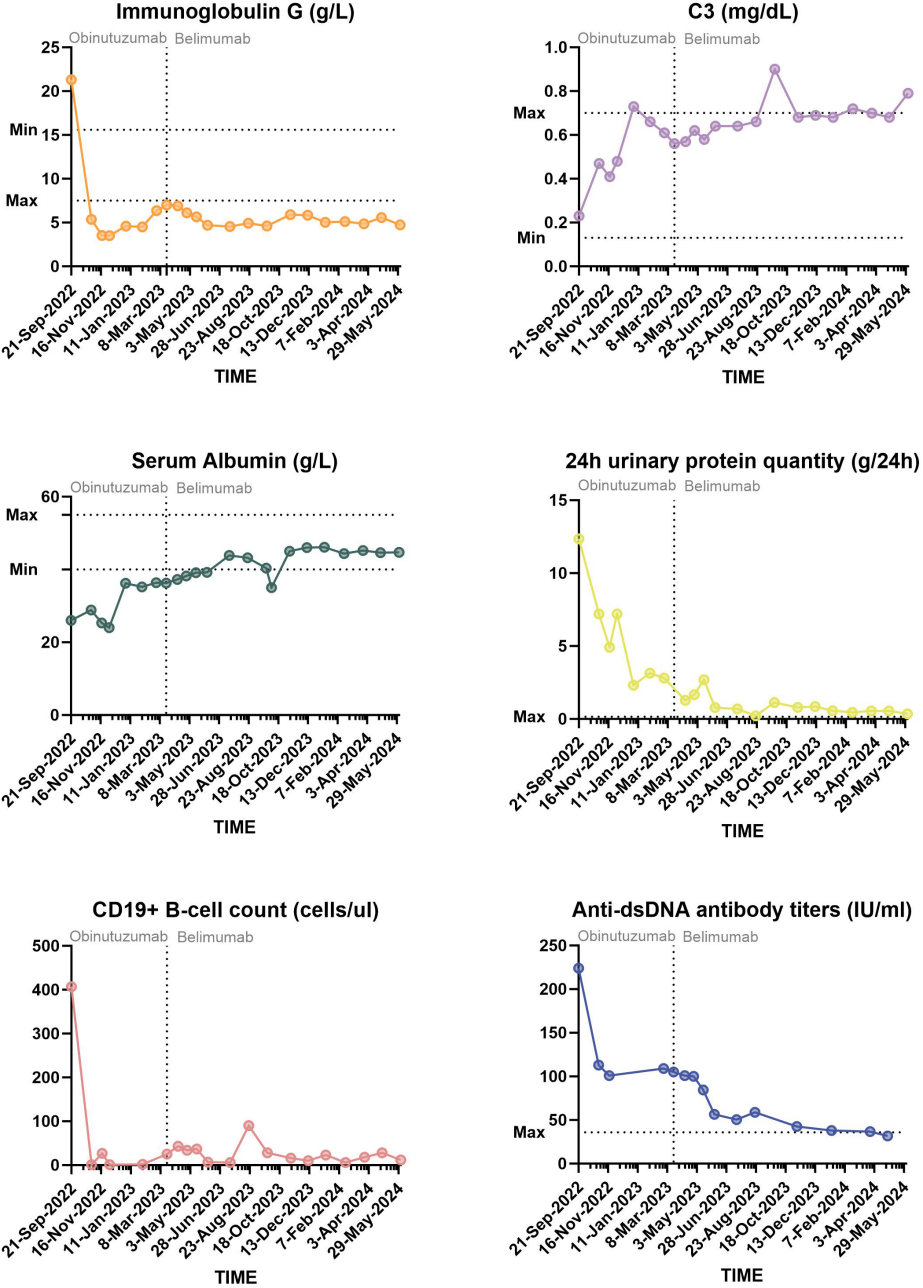
Diagram of the changes in the SLE major activity index over 18 months of the follow-up period.

**Figure 3:**
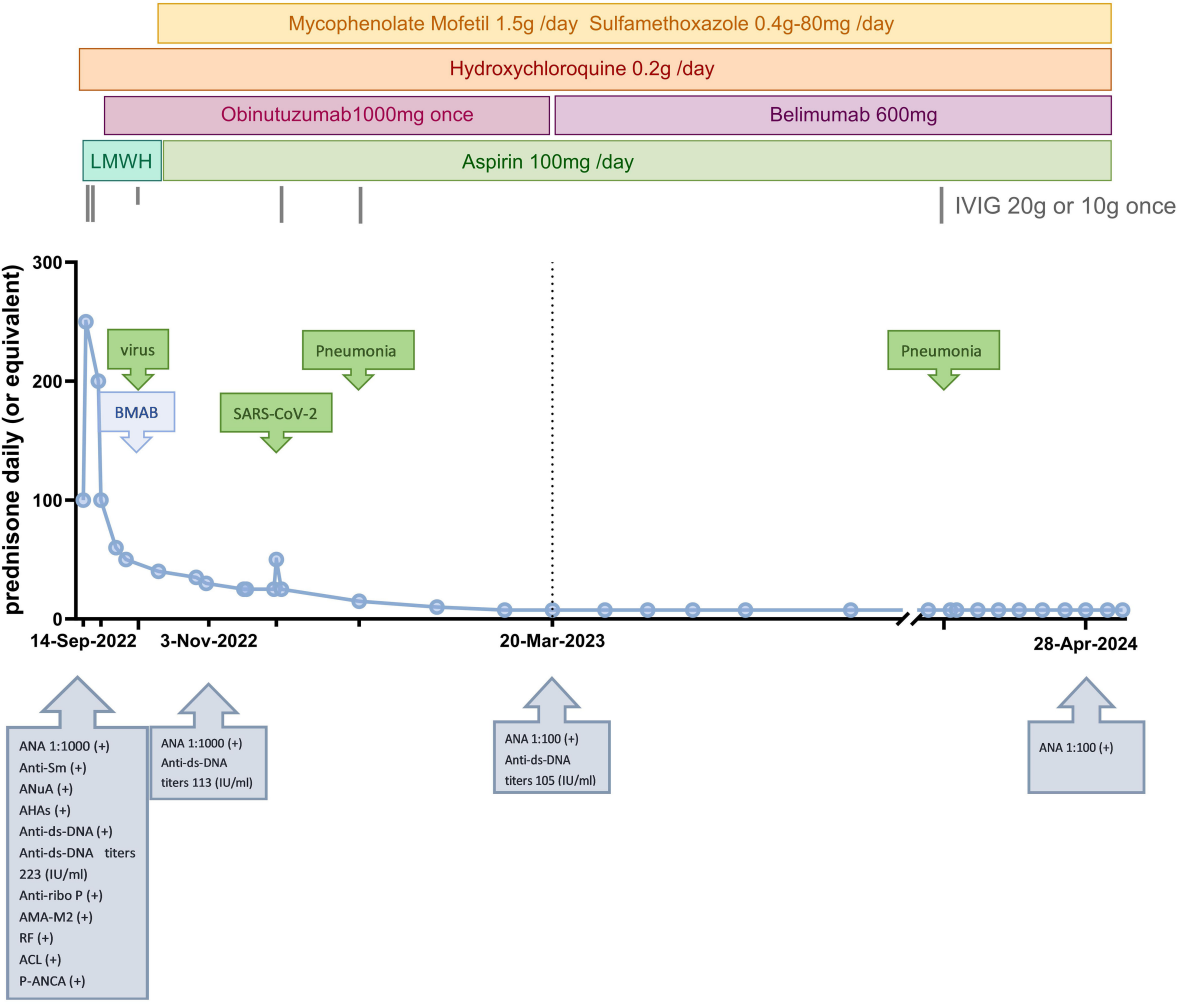
Clinical course since hospitalization. BMAB, bone marrow aspirator and biopsy; IVIG, intravenous immunoglobulin; LMWH, low-molecular weight heparin; ANuA, anti-nucleosome antibody; AHA, anti-histone antibody; anti-ribo P, anti-ribosomal P protein antibody; AMA-M2, anti-mitochondrial antibody M2; RF, rheumatoid factor; ACL, anti-cardiolipin antibody; P-ANCA, p-type anti-neutrophil cytoplasmic antibody.

During the first 6 months of follow-up, the patient developed two more infections; one was bacterial pneumonia, and the other was SARS-CoV-2. Owing to her consistently lower than normal IgG levels and repeated infections, she did not receive the second dose of obinutuzumab. On 3 March 2023, her prednisone dose was reduced to 10 mg/day. Numerous immune indices in the re-examination group tended to deteriorate again (**Figure 2**). The lowest level of 24UTP was 2.32 g, indicating a positive response to prior treatment for LN but not complete remission. Faced with these challenges, we decided to explore another biologic, namely, belimumab. Since belimumab primarily targets BAFF, it may not reduce antibody production as significantly as treatments that deplete B cells do, thereby helping to preserve the body’s defences against infection. Over the next 12 months, the patient began receiving regular doses of 600 mg each. Her IgG levels remained low, and in September 2023, she experienced another episode of pneumonia, which was cured by anti-infective treatment. Nevertheless, her SLE condition was stable; her laboratory indicators changed slowly but steadily improved. At the most recent re-examination on 30 April 2024, all of her autoantibodies, including her anti–ds-DNA antibody titre, were negative, and her 24UTP level was 0.36 g, indicating both clinical and serological remission. The patient’s clinical course is summarized in **Figure 3**. The corresponding laboratory changes over 18 months are shown in the **Figure 2** (as of 24 June 2024).

## Discussion

A number of therapies are available that target B cells in the treatment of SLE (9). Strategies have included direct B-cell killing, modulation of B-cell function, inhibition of molecules essential for B-cell growth and survival, and acceleration of autoantibody clearance (1). The concept of biologic combination therapy was not first proposed. A phase 2, single-arm, proof-of-concept study was pioneering in treating 15 patients with severe, refractory SLE using a combination of rituximab and belimumab, with a 2-year follow-up. These patients exhibited a substantial decrease in autoantibody levels and a marked reduction in circulating B cells (10). Preliminary evidence has subsequently been obtained in RCTs for the effectiveness of a combination regimen of rituximab followed by belimumab for SLE treatment. The BEAT-LUPUS study revealed that, compared with rituximab followed by placebo, rituximab followed by belimumab significantly reduced serum IgG anti–ds-DNA antibody levels from baseline (70% vs. 30%, *P* < 0.001), as well as the risk of severe recurrence (hazard ratio 0.27, *P* = 0.033) (7). However, in another RCT, the clinical efficacy of rituximab and cyclophosphamide in combination with belimumab did not improve compared to that of a therapeutic strategy of B-cell depletion alone in LN patients (52% vs. 41%, *P* = 0.452) (11). Therefore, the search for suitable subjects, timing, or combinations is remains underway.

We reviewed the literature with a focus on obinutuzumab and belimumab, and no similar previously published reports were found. Data on rituximab explain why there is a positive response to combination therapy (7, 12). After rituximab treatment depletes B cells, BAFF levels may increase, which may limit the efficacy of initial B-cell clearance. The use of belimumab to inhibit BAFF may help further control SLE. In addition, there are several key differences between obinutuzumab and rituximab (8). In particular, the glycosylation of obinutuzumab in the Fc segment enhances its affinity for immune effector cells, which can enhance antibody-dependent cell-mediated cytotoxicity (ADCC) and antibody-dependent cell-mediated phagocytosis. In *in vitro* studies, the ADCC activity induced by obinutuzumab was 35 to 100 times greater than that induced by rituximab. These differences give obinutuzumab some potential advantages.

During more than 18 months post-obinutuzumab follow-up, the patient had consistently low IgG levels. Although the specific data on obinutuzumab are not clear, studies have shown that rituximab use may lead to increased infection in patients with low IgG levels (13). In fact, the patient suffered three respiratory infections in 6 months, leading to repeated delays in the sequencing of obinutuzumab. We wondered whether the SLE therapeutic strategy had to follow the same frequency as the obinutuzumab regimen for lymphoma in the context of significantly low IgG and B-cell counts, notwithstanding the immune escape phenomenon. In addition, to date, a long-term regimen following obinutuzumab in SLE patients has not been established. Therefore, it is necessary to consider the possibility of using belimumab as a long-term maintenance regimen after obinutuzumab. We observed that during the first 6 months of treatment, the patient achieved a partial response. Therefore, it is necessary to maintain or repeat therapy targeting B cells. Depletion of B cells affect the production of antibodies, which may lead to a decline in immune defence capabilities. Belimumab primarily affects the survival and proliferation of self-reactive B cells rather than broadly depleting all B cells; thus, its impact on immune defence may be less significant than that of obinutuzumab (14). Interestingly, COVID-19 was more common in a small group of SLE patients who received belimumab treatment (15, 16). Therefore, sequential belimumab therapy may be a complementary option for patients who already have frequent infections (14). Notably, as a biologic that targets B cells, belimumab may also increase the risk of infection, especially when it is used in combination with other immunosuppressive drugs. We noted that the patient experienced pneumonia again while on belimumab, but it occurred less frequently than in the past.

After treatment with belimumab following obinutuzumab administration, the patient successfully achieved a complete clinical response, and all specific autoantibodies tested negative. According to the European League Against Rheumatism guidelines on the management of SLE, such treatment aims to control disease activity, prevent disease recurrence and organ damage, optimize health-related quality of life, and ultimately extend the life of patients (17). Notably, the guidelines do not make specific provisions on serological standards. We chose to add MMF to the biologic regimen, because it is a classical treatment of choice for LN and is the background drug of the NOBILITY clinical trial. However, in terms of the therapeutic effect of MMF, achieving completely negative serological results is unrealistic. Indeed, in the traditional treatment model, the requirement to achieve both clinical and serological criteria may also be an unreachable goal. However, with the advent of the era of targeted therapy, double clinical and serological standards are gradually becoming possible for SLE treatment (18). Our patient achieved thorough serological compliance with the long-term sequential use of belimumab after obinutuzumab and achieved a balance between clinical efficacy and safety.

We are well aware of the limitations of the present case. First, from a diagnostic point of view, the failure to obtain renal pathology is regrettable. Owing to the large amount of ascites in the patient’s abdominal cavity at that time, renal puncture was delayed. Prior to discharge, we obtained informed consent for her renal puncture but cancelled it again because of her severe cough. Therefore, the diagnosis of LN in this case was based on the 1997 ACR criteria rather than pathological findings (19), which is a major flaw. Second, the contribution of IVIG, MMF, and other treatments to clinical efficacy remains unclear. Third, follow-up regimens for drug withdrawal in patients remain challenging and require longer term observation. The patient, who said she felt she was recovering well, stated that she should have cooperated with physicians earlier before severe internal damage occurred to avoid extremely difficult treatment later. This patient provided written informed consent for case publication. Ethical approval was obtained from the Medical Ethics Committee of the Second Hospital of Jiaxing (2023-CA06). In conclusion, we believe that there is reason to be optimistic. This novel combination regimen can potentially be developed as a therapeutic strategy. Additional research, particularly RCTs, will provide a definitive demonstration of the efficacy of this sequential therapy.

## Data Availability

The original contributions presented in the study are included in the article/supplementary material. Further inquiries can be directed to the corresponding author.

## References

[1] Krustev E Clarke AE Barber MRW. B cell depletion and inhibition in systemic lupus erythematosus. Expert Rev Clin Immunol. (2023) 19:55–70. doi: 10.1080/1744666X.2023.2145281 36342225

[2] Iwata S Hajime Sumikawa M Tanaka Y. B cell activation via immunometabolism in systemic lupus erythematosus. Front Immunol. (2023) 14:1155421. doi: 10.3389/fimmu.2023.1155421 37256149 PMC10225689

[3] Möckel T Basta F Weinmann-Menke J Schwarting A. B cell activating factor (BAFF): Structure, functions, autoimmunity and clinical implications in Systemic Lupus Erythematosus (SLE). Autoimmun Rev. (2021) 20:102736. doi: 10.1016/j.autrev.2020.102736 33333233

[4] Furie RA Aroca G Cascino MD Garg JP Rovin BH Alvarez A. B-cell depletion with obinutuzumab for the treatment of proliferative lupus nephritis: a randomised, double-blind, placebo-controlled trial. Ann Rheum Dis. (2022) 81:100–7. doi: 10.1136/annrheumdis-2021-220920 PMC8762029 34615636

[5] Merrill JT Neuwelt CM Wallace DJ Shanahan JC Latinis KM Oates JC. Efficacy and safety of rituximab in moderately-to-severely active systemic lupus erythematosus: the randomized, double-blind, phase II/III systemic lupus erythematosus evaluation of rituximab trial. Arthritis Rheum. (2010) 62:222–33. doi: 10.1002/art.27233 PMC4548300 20039413

[6] Rovin BH Furie R Latinis K Looney RJ Fervenza FC Sanchez-Guerrero J. Efficacy and safety of rituximab in patients with active proliferative lupus nephritis: the Lupus Nephritis Assessment with Rituximab study. Arthritis Rheum. (2012) 64:1215–26. doi: 10.1002/art.34359 22231479

[7] Shipa M Embleton-Thirsk A Parvaz M Santos LR Muller P Chowdhury K. Effectiveness of belimumab after rituximab in systemic lupus erythematosus: A randomized controlled trial. Ann Intern Med. (2021) 174:1647–57. doi: 10.7326/M21-2078 34698499

[8] Freeman CL Sehn LH. A tale of two antibodies: obinutuzumab versus rituximab. Br J Haematol. (2018) 182:29–45. doi: 10.1111/bjh.15232 29741753

[9] Wise LM Stohl W. Belimumab and rituximab in systemic lupus erythematosus: A tale of two B cell-targeting agents. Front Med (Lausanne). (2020) 30:303. doi: 10.3389/fmed.2020.00303 PMC7338653 32695790

[10] Kraaij T Arends EJ van Dam LS Kamerling SWA van Daele PLA Bredewold OW. Long-term effects of combined B-cell immunomodulation with rituximab and belimumab in severe, refractory systemic lupus erythematosus: 2-year results. Nephrol Dial Transplant. (2021) 36:1474–83. doi: 10.1093/ndt/gfaa117 PMC8311580 32591783

[11] Atisha-Fregoso Y Malkiel S Harris KM Byron M Ding L Kanaparthi S. Phase II randomized trial of rituximab plus cyclophosphamide followed by belimumab for the treatment of lupus nephritis. Arthritis Rheumatol. (2021) 73:121–31. doi: 10.1002/art.41466 PMC7839443 32755035

[12] Gualtierotti R Borghi MO Gerosa M Schioppo T Larghi P Geginat J. Successful sequential therapy with rituximab and belimumab in patients with active systemic lupus erythematosus: a case series. Clin Exp Rheumatol. (2018) 36:643–7. 29533753

[13] Barber MRW Clarke AE. Systemic lupus erythematosus and risk of infection. Expert Rev Clin Immunol. (2020) 16:527–38. doi: 10.1080/1744666X.2020.1763793 32478627

[14] Kirou KA Dall Era M Aranow C Anders HJ. Belimumab or anifrolumab for systemic lupus erythematosus? A risk-benefit assessment. Front Immunol. (2022) 13:980079. doi: 10.3389/fimmu.2022.980079 36119023 PMC9472122

[15] Ugarte-Gil MF Alarcon GS Izadi Z Duarte-Garcıa A Reategui-Sokolova C Clarke AE. Characteristics associated with poor COVID-19 outcomes in individuals with systemic lupus erythematosus: data from the COVID-19 global rheumatology alliance. Ann Rheumatol Dis. (2022) 81:970–8. doi: 10.1136/annrheumdis-2021-221636 PMC8882632 35172961

[16] Mehta P Gasparyan AY Zimba O Kitas GD. Systemic lupus erythematosus in the light of the COVID-19 pandemic: infection, vaccination, and impact on disease management. Clin Rheumatol. (2022) 81:970–8. doi: 10.1007/s10067-022-06227-7 PMC9152659 35639259

[17] Fanouriakis A Kostopoulou M Alunno A Aringer M Bajema I Boletis JN. 2019 update of the EULAR recommendations for the management of systemic lupus erythematosus. Ann Rheum Dis. (2019) 78:736–45. doi: 10.1136/annrheumdis-2019-215089 30926722

[18] Emmi G Bettiol A Palterer B Silvestri E Vitiello G Parronchi P. Belimumab reduces antiphospholipid antibodies in SLE patients independently of hydroxychloroquine treatment. Autoimmun Rev. (2019) 18:312–4. doi: 10.1016/j.autrev.2018.11.002 30639638

[19] Hochberg MC. Updating the American College of Rheumatology revised criteria for the classification of systemic lupus erythematosus. Arthritis Rheum. (1997) 40:1725. doi: 10.1002/art.1780400928 9324032

